# CRISPR gene editing to improve crop resistance to parasitic plants

**DOI:** 10.3389/fgeed.2023.1289416

**Published:** 2023-10-25

**Authors:** Min-Yao Jhu, Evan E. Ellison, Neelima R. Sinha

**Affiliations:** ^1^ Crop Science Centre, Department of Plant Sciences, University of Cambridge, Cambridge, United Kingdom; ^2^ Department of Plant Biology, University of California, Davis, Davis, CA, United States

**Keywords:** CRISPR, parasitic plants, haustorium, resistance, defence, gene editing, inducible defence responses, cell-type specific

## Abstract

Parasitic plants pose a significant threat to global agriculture, causing substantial crop losses and hampering food security. In recent years, CRISPR (Clustered Regularly Interspaced Short Palindromic Repeats) gene-editing technology has emerged as a promising tool for developing resistance against various plant pathogens. Its application in combating parasitic plants, however, remains largely unexplored. This review aims to summarise current knowledge and research gaps in utilising CRISPR to develop resistance against parasitic plants. First, we outline recent improvements in CRISPR gene editing tools, and what has been used to combat various plant pathogens. To realise the immense potential of CRISPR, a greater understanding of the genetic basis underlying parasitic plant-host interactions is critical to identify suitable target genes for modification. Therefore, we discuss the intricate interactions between parasitic plants and their hosts, highlighting essential genes and molecular mechanisms involved in defence response and multilayer resistance. These include host resistance responses directly repressing parasitic plant germination or growth and indirectly influencing parasitic plant development via manipulating environmental factors. Finally, we evaluate CRISPR-mediated effectiveness and long-term implications for host resistance and crop improvement, including inducible resistance response and tissue-specific activity. In conclusion, this review highlights the challenges and opportunities CRISPR technology provides to combat parasitic plants and provides insights for future research directions to safeguard global agricultural productivity.

## 1 Introduction

Plant pests and pathogens significantly threaten global food security, causing substantial yield losses (Savary et al., 2019). Climate change exacerbates the issue by altering pathogen assemblages (Chaloner et al., 2021). Efficient plant disease management is essential to sustainably meet global food demand. Current disease management methods include chemical control, which is efficient but can have adverse environmental impacts and promotes resistance (Yin and Qiu, 2019). On the other hand, biological control, while more environmentally friendly, often exhibits relatively limited consistency and cost-effectiveness (Gerbore et al., 2014). However, successful examples of utilizing biological controls and natural resistance varieties have demonstrated their potential in effectively managing plant pests and diseases (Sauerborn et al., 2007; Gerbore et al., 2014). This indicates that leveraging host resistance could offer a promising and more sustainable alternative solution.

Therefore, harnessing knowledge about plant-pathogen interactions and defence responses is crucial for developing successful disease management strategies (Veillet et al., 2020). Developing disease-resistant crops relies on comprehending multi-dimensional defence mechanisms, including pattern-triggered immunity (PTI) and effector-triggered immunity (ETI), to combat invading pathogens (Langner et al., 2018). Introducing host resistance through conventional breeding is hindered by linkage drag and limited genetic diversity within elite germplasm (Tester and Langridge, 2010). Mutation breeding introduces variation but also genome-wide undesired mutations (Toker et al., 2007). Genome editing, particularly CRISPR-Cas, enables precise gene modifications without off-target detrimental effects (Menz et al., 2020).

In this review, we summarise the role of CRISPR in developing resistance against parasitic plants, outlining its improvements and applications against pathogens. Understanding the genetic basis of plant-host interactions is vital for targeted gene modification. We explore essential genes and mechanisms for defence and resistance, evaluating CRISPR’s effectiveness in enhancing crop resistance. We outline the challenges and opportunities of CRISPR technology for safeguarding agricultural productivity.

## 2 CRISPR editing tools and recent technological advances

Applications in plant biology have been no exception to the promise of targeted genome manipulation provided by CRISPR/Cas systems (Gao, 2021). While some of the earliest examples of CRISPR/Cas utility in plant biology were gene knockouts in model organisms, the technology has now been expanded to a wide variety of applications including large-scale editing screens, base editing, targeted insertions, and transcriptomic and epigenomic modifications (Gaillochet et al., 2021; Pan et al., 2021; Ren et al., 2021; Zong et al., 2022). In parallel, improvements have been made in the delivery of CRISPR/Cas and other plant genome engineering reagents to plant cells, particularly for non-model and crop species (Ellison et al., 2020; Maher et al., 2020; Che et al., 2022; Demirer et al., 2023). Together, advancements in genome editing technology with efficient delivery of reagents provide great promise for gene discovery and functional genome modification.

RNA guided endonuclease systems, such as CRISPR/Cas, provide incredible precision for modifying specific targets in the genome. CRISPR systems utilize a guide RNA (gRNA) comprised of a constant repeat sequence and spacer sequence specific to a desired target site (Jinek et al., 2012). The only requirement for this target is an adjacent protospacer adjacent motif (PAM), which for *S. pyogenes* Cas9 (SpCas9) consists of a simple 5′-NGG-3′ sequence (Jinek et al., 2012). Minimal target sequence requirements, ease in reagent design, and robust cleavage has quickly established CRISPR as a highly effective tool for targeted genetic modification.

Many examples of CRISPR application in plants prioritize targeting protein coding sequences, using indels to induce a frameshift mutation (Zsögön et al., 2018). This approach has been employed for large scale screens in which dozens to thousands of unique mutants are generated to uncover novel gene function and epistasis (Gaillochet et al., 2021). The adaptation of CRISPR systems from other species, such as CRISPR/Cas12 from Lachnospiraceae bacterium ND 2006 which recognizes TTTV (V = A, C, and G) PAM sequences, has provided greater flexibility in target site requirements (Zhang et al., 2021). Greater precision in modification type is provided by base editing via cytidine or adenine deaminases fused to Cas9 nickases which can be exploited for specific nucleotide or amino acid changes (Ren et al., 2021). This precision is expanded by the recent development of prime editors for targeted sequence modification, deletion, or insertion (Zong et al., 2022). In other applications, CRISPR is used to modify or disrupt noncoding or regulatory elements resulting in quantitative variation (Rodríguez-Leal et al., 2017). Modifications to gene regulation, however, are not limited to genetic changes. By using a catalytically inactive Cas protein tagged with transcriptional or epigenomic regulators, gene expression can be regulated in a target-specific manner without inducing double-stranded breaks (Pan et al., 2021). We recommend a recent review for a more comprehensive discussion on recent developments in CRISPR/Cas plant genome engineering reagents (Capdeville et al., 2023).

## 3 CRISPR applications in disease and parasite resistance

Recent advancements in genome editing technology provide powerful tools to address various agricultural challenges, including creating disease and pest-resistant crop lines (Langner et al., 2018; Karmakar et al., 2022). CRISPR/Cas systems have demonstrated remarkable efficiency in combatting virus infections, as well as fungal and bacterial diseases across diverse plant species (Boubakri, 2023). This versatile technology holds immense promise for revolutionising agricultural practices and bolstering crop resilience against pathogenic threats.

Engineering host resistance in plants has long been anchored in the classical “gene for gene” hypothesis. This principle revolves around the interaction between host R (resistance) genes and pathogen Avr (avirulence) genes, determining the outcome of resistance or disease occurrence. One approach for broad-spectrum resistance is through the modification of R genes by CRISPR/Cas reagents (Dangl et al., 2013). Precisely mutating the leucine-rich repeat (LRR) domain within R genes enables alterations in elicitor recognition specificity and confers resistance against diverse pathogens. However, relying solely on a single R gene for resistance may prove inadequate due to pathogen mutations that might enable them to circumvent specific resistance mechanisms, necessitating the exploration of alternative strategies. Concurrently, host susceptibility (S) genes are potential targets for engineering host resistance (van Schie and Takken, 2014). CRISPR/Cas editing of S genes results in durable, broad-spectrum resistance against fungal and bacterial pathogens.

In summary, the transformative potential of CRISPR/Cas tools in engineering disease resistance in plants presents exciting opportunities in agricultural research. While several review articles have discussed the application of CRISPR in plant disease resistance (Langner et al., 2018; Yin and Qiu, 2019; Boubakri, 2023), it is crucial to recognise that plant pathogens, such as viruses, bacteria, and fungi, are not the sole threats to food security. Parasitic plants also significantly impact agricultural productivity worldwide (Jhu and Sinha, 2022). According to a stochastic model that has been published, it is projected that the yearly economic losses attributed to all parasitic weeds will likely reach approximately US $200 million, with an annual rise of roughly US $30 million (Rodenburg et al., 2016). Compared to abundant studies on plant pathogens, research and discussion on host resistance mechanisms to combat parasitic plants are relatively limited. The application of CRISPR technologies to improve crops’ defence against parasitic plants is still in its early stages and lacks a systematic review. Therefore, this review will focus on the importance and significance of utilising CRISPR to resist parasitic plants, highlighting past successful examples and proposing potential future research directions to foster resilient and sustainable crop protection measures.

## 4 Notorious parasitic weeds and global food security

**FIGURE 1:**
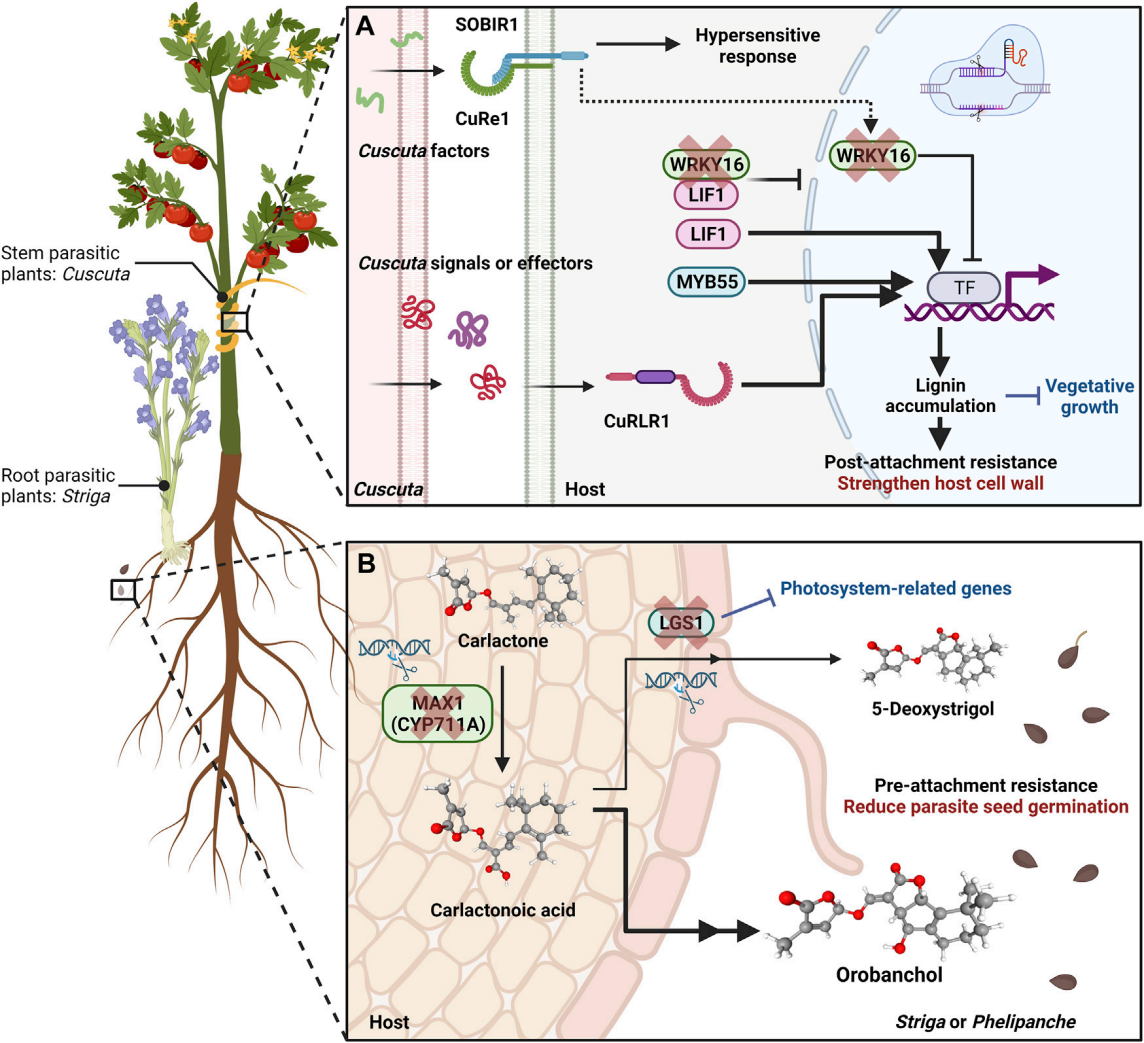
Utilizing CRISPR Techniques to Enhance Pre-attachment and Post-attachment Defence Mechanisms against Parasitic Plants. **(A)** Overview of a CRISPR-based approach to reinforce the host plant’s resistance mechanisms against stem parasitic *Cuscuta* species during and after attachment. The cellular receptor CUSCUTA RECEPTOR 1 (CuRe1) is a leucine-rich repeat (LRR) receptor-like protein (RLP) responsible for recognizing *Cuscuta*-derived factors at the cell surface (Hegenauer et al., 2016; Hegenauer et al., 2020). Teaming up with the coreceptor SlSOBIR1, this recognition event initiates downstream defensive reactions, including hypersensitive responses. In resistant tomato cultivars, the *Cuscuta* R-gene for lignin-based resistance 1 (CuRLR1) is an N-terminal coiled-coil (CC)-nucleotide-binding site (NBS)-LRR protein (Jhu et al., 2022a). CuRLR1 might be involved in sensing specific signalling pathways or even function as a receptor for identifying unknown signals or effectors produced by *Cuscuta*. Activation of CuRLR1 sets off subsequent signalling sequences, leading to the activation of genes participating in the lignin biosynthesis pathway. Consequently, there is a buildup of lignin in the cortex region of the tomato stem, acting as a physical barrier to hinder haustorium penetration. Transcription factors like Lignin Induction Factor 1 (LIF1; an AP2-like transcription factor) and MYB55 positively regulate enhanced resistance based on host lignin. Conversely, WRKY16, which experiences upregulation upon infestation by *Cuscuta campestris*, plays a critical role as a negative regulator of lignin production and the function of LIF1. Based on previous research, one hypothesis suggests that WRKY16 acts as a connecting link (indicated by a dashed arrow) between CuRe1 and the lignification response. By employing CRISPR technology to target and knockout WRKY16 precisely, a sustained accumulation of lignin is achieved, thereby reinforcing the plant’s resilience against *C. campestris*. **(B)** Overview of CRISPR Applications for Reinforcing Pre-attachment Resistance by Impeding Seed Germination of Root Parasitic Plants. The biosynthesis of strigolactones (SLs), orchestrated by the carotenoid pathway involving genes like *More Axillary Growth 1* (*MAX1*), is a pivotal mechanism explored for enhancing pre-attachment resistance. The *MAX1* genes encode cytochrome P450 monooxygenases of the CYP711A subfamily, acting as carlactone (CL) oxidases responsible for converting CL into carlactonoic acid. CRISPR-based knockout generated max1 mutant lines demonstrate heightened resilience against the root parasitic plant *Phelipanche aegyptiaca*. This resilience is attributed to reduced SL levels due to max1 mutant. *LOW GERMINATION STIMULANT 1* (*LGS1*), encoding a sulfotransferase enzyme, is pivotal in SL biosynthesis. In susceptible sorghum host plants, the principal SL in root exudates is 5-deoxystrigol, a potent stimulant for root parasitic plant Striga seed germination. In contrast, orobanchol, an SL with an opposing stereochemistry to 5-deoxystrigol, fails to induce *Striga* seed germination. By leveraging CRISPR technology, targeted mutations in *LGS1* facilitate a shift in the dominant SL composition within host plant root exudates. This composition changes from 5-deoxystrigol to orobanchol, significantly reducing parasite seed germination rates. Consequently, these altered root exudates enhance pre-attachment resistance in the host plants. The three-dimensional structural representations of carlactone, carlactonoic acid, orobanchol, and 5-deoxystrigol are from PubChem. Text highlighted in red indicates the key reinforced resistance responses, while text highlighted in blue signifies the potential trade-off side effects associated with constitutively activated resistance responses. This figure was created with https://www.biorender.com/.

Parasitic plants pose a significant risk to food security globally, approximately affecting millions of hectares of croplands and targeting vital cereal crops and vegetables (Lanini and Kogan, 2005; Ejeta, 2007). These parasitic weeds develop specialised organs, haustoria, to invade host vascular systems and hijack water and nutrients (Yoshida et al., 2016), leading to substantial reductions in agricultural productivity and, in some cases, complete crop failure (Lanini and Kogan, 2005; David et al., 2022). Based on the host tissue invaded, parasitic weeds can be classified as stem or root parasites (Yoshida et al., 2016). Host-dependence further categorises them into obligate hemiparasitic, facultative hemiparasitic, or holoparasitic. A well-known example of stem holoparasitic plants is the *Cuscuta* species (dodders) (Figure 1), which parasitises numerous critical vegetable and fruit crops. Conversely, root hemiparasitic plants, like the *Striga* species, commonly known as witchweed (Figure 1), parasitise a broad spectrum of cereal crops. More detailed classification descriptions have been well discussed in previous review articles (Yoshida et al., 2016). These diverse classifications highlight the complexity of parasitic weed interactions with host plants and ecosystems. Controlling parasitic plants is challenging due to their well-adapted life cycles, high seed production, and genetic diversity. For example, *Striga* can produce up to 0.5 million seeds per plant, with seeds remaining viable in the soil for extended periods (David et al., 2022). Their ability to disperse seeds widely and adapt to various environments makes eradication problematic.

Various methods have been attempted to manage parasitic plant infestations, including agricultural practices, chemical or bioinoculant applications, and host resistances (Sauerborn et al., 2007). However, none of these methods alone provides a sustainable, long-term solution. Conventional practices like hand weeding and crop rotation have shown limited success (Kanampiu et al., 2018), often due to factors such as continuous monocropping, which create favourable conditions for the spread of parasitic plants. For a more effective and sustainable approach to controlling Striga, utilising multiple-layer defence and resistance mechanisms and integrating parasitic plant-resistant or -tolerant cultivars with current agricultural practice can provide more promising results (Abdullahi et al., 2022).

## 5 CRISPR applications in enhancing resistance against parasitic plants

### 5.1 Identifying targets for CRISPR: pre-attachment and post-attachment resistance

Understanding how host plants defend against parasitic plants is crucial for effectively utilizing gene editing to enhance host resistance. Recent research has highlighted similar host-parasitic plant defence response to interactions seen in other host-pathogen relationships (Fishman and Shirasu, 2021; Jhu and Sinha, 2022). The initial response involves pathogen-triggered immunity (PTI), activating physical and biochemical defences within host plant cells upon detecting parasite presence. However, parasitic plants can counter PTI by introducing effectors into host cells, thus promoting parasitism (Li and Timko, 2009; Su et al., 2020). Should the host possess resistance, this leads to effector-triggered immunity (ETI), causing programmed cell death and thwarting further parasite development.

Host resistance mechanisms can be divided into pre-attachment and post-attachment categories based on whether these defences occur before or after parasitic plants establish themselves on hosts (Fishman and Shirasu, 2021; Jhu and Sinha, 2022). The strategies of pre-attachment and post-attachment resistance against root parasitic plants are briefly introduced in the following sections. More comprehensive insights into the underlying mechanisms can be found in prior review publications (Fishman and Shirasu, 2021; Jhu and Sinha, 2022).

### 5.2 CRISPR applications in enhancing pre-attachment resistance

Pre-attachment resistance encompasses a range of strategies employed by host plants to prevent the attachment and invasion of parasitic plants before direct contact occurs. These mechanisms include inhibiting the germination of parasitic plant seeds. Strigolactones (SLs), a class of plant hormones, play a crucial role in triggering the germination of root parasitic plants in the family Orobanchaceae (Yoneyama et al., 2010) and signalling mycorrhizal associations in soil (Waters et al., 2017; Kodama et al., 2022). Various types of SLs have been identified as inducers for parasitic plant growth. For instance, mutations affecting SL production or composition in Striga species lead to diminished germination rates (Gobena et al., 2017).

In addition to inhibiting parasite seed germination, some host plants release toxic compounds through their root exudates, hampering the development of parasitic plant seedlings (Serghini et al., 2001; Echevarría-Zomeño et al., 2006). For example, certain resistant sunflower varieties produce toxic coumarins that impede *Orobanche* development (Serghini et al., 2001). On the other hand, some hosts interfere with haustorium initiation: a vital first step for establishing a connection between host and parasite. Similarly, specific sorghum variants disrupt the haustorium formation of *Striga asiatica*, potentially through the release of inhibitory substances in root exudates (Rich et al., 2004). These diverse defence strategies of host plants against parasitic plants offer promising avenues and targets for CRISPR approaches in tackling parasitic plant infestations and advancing agricultural sustainability.

In recent studies, genetic manipulation techniques such as CRISPR-Cas9 have been employed to target genes responsible for strigolactone biosynthesis and parasitism, resulting in resistance against parasitic plants in crops respectively (Bellis et al., 2020; Bari et al., 2021). For example, mutations affecting the *LOW GERMINATION STIMULANT 1* (*LGS1*) gene within resistant Sorghum plants bring changes in the composition of strigolactones (SLs) found in root exudates, resulting in a decrease in the stimulatory impact on Striga germination (Figure 1) (Gobena et al., 2017). *LGS1* encodes a sulfotransferase enzyme, and its functional loss leads to a shift from the potent *Striga* germination stimulant, 5-deoxystrigol, to orobanchol, an SL with differing stereochemistry (Figure 1) (Gobena et al., 2017).

However, these alterations in SLs have broader effects. Recent CRISPR/Cas9 edited sorghum experiments emphasize that the benefits of *LGS1*-based resistance are influenced by parasite genotype and environmental conditions (Bellis et al., 2020). *LGS1* knockout lines may exhibit increased susceptibility to *S. hermonthica* genotypes sensitive to orobanchol. Additionally, *LGS1* mutant lines demonstrate a trade-off of diminished expression of photosystem-related genes (Bellis et al., 2020). The systemic reduction in these genes within *LGS1* knockout lines corresponds to the known role of SLs in enhancing light harvesting (Mayzlish-Gati et al., 2010). Consequently, relying solely on these CRISPR knockout lines and widespread deployment could present challenges in sorghum cultivation.

Similarly, SL biosynthesis is also a target for CRISPR/Cas mediated resistance. SLs are produced through the carotenoid pathway involving *Carotenoid Cleavage Dioxygenase* (*CCD*) *7*, *CCD8*, and *More Axillary Growth 1* (*MAX1*) genes (Alder et al., 2012; Seto et al., 2014). Through CRISPR/Cas9-mediated gene knockout in tomato, MAX1 disruption renders resistance against the root parasitic weed *Phelipanche aegyptiaca* (Bari et al., 2021) (Figure 1). These *MAX1*-Cas9 mutant lines demonstrate heightened resistance to *P. aegyptiaca* due to reduced levels of SL (specifically orobanchol). However, this genetic alteration influenced the expression of the carotenoid biosynthesis gene phytoene desaturase-1 (*PDS1*) and overall carotenoid levels compared to their wild-type counterparts. Noteworthy, *MAX1*-Cas9 plants exhibited morphological shifts, such as increased growth of axillary buds, decreased plant height, and the emergence of adventitious roots, diverging from the wild type (Bari et al., 2021).

The intricate mechanisms underlying pre-attachment resistances remain largely unexplored. The identification of Striga resistance genes plays a vital role in the development of genotypes boasting lasting resistance. Fortunately, recent studies have leveraged population structure analysis and genome-wide association studies (GWAS) to pinpoint promising candidates (Adewale et al., 2020; Kavuluko et al., 2021). The application of CRISPR knockout techniques can help dissect the function of these candidate genes and pathways. However, it is essential to acknowledge that genetically modified plants generated through CRISPR knockout may face growth/defence trade-offs. Relying solely on CRISPR knockout lines could potentially pose agricultural challenges. Therefore, to tackle this concern, the integration of advanced CRISPR technologies with meticulous regulation mechanisms like inducible systems or tissue-specific expression becomes pivotal for effectively deploying this approach in agriculture without compromising yield potential.

### 5.3 CRISPR applications in enhancing post-attachment resistance

Following attachment, post-attachment resistance unfolds as a plant’s defensive strategy, activated upon detection of parasitic plants affixed to the host. This defence repertoire encompasses various mechanisms, such as hypersensitive responses (HRs), hormone-driven signalling pathways, fortification of cell walls, and accumulation of defensive secondary metabolites (Fishman and Shirasu, 2021; Jhu and Sinha, 2022).

Among these post-attachment resistance responses, modifying cell walls has been prominently observed and reported in prior research as a crucial strategy. Various host plants resistant to root and stem parasitic plants have harnessed this mechanism (Fishman and Shirasu, 2021; Jhu and Sinha, 2022). For instance, investigations reveal that specific Heinz tomato cultivars exhibiting resistance manifest inducible lignin-based defence responses upon encountering the stem parasitic plant *Cuscuta campestris* (Jhu et al., 2022a). Using CRISPR to target and knock out the key negative regulator of this lignin-based response yields a state of constant lignin accumulation, bolstering the host plants’ resilience against *C. campestris* (Figure 1). However, this fortification comes at the expense of compromised vegetative growth (Jhu et al., 2022a).

Further exploration of different mechanisms in post-attachment resistances and their integration into plant genetic engineering is essential. While identifying pivotal elements within defence mechanisms marks progress, it is evident that this information alone falls short. It is imperative to delve into the facilitators of inducible responses and strategically integrate these systems—encompassing potential promoters, regulators, and receptors—into plant genetic engineering (Zaidi et al., 2020).

## 6 Discussions and future perspectives

Many current and future applications of CRISPR-mediated editing prioritize induction of genomic modifications followed by segregating away lines with active CRISPR systems. This is valuable when considering near-term agricultural incorporation and public acceptance (Pixley et al., 2022). While CRISPR-mediated gene modifications provide a valuable resource, it is crucial to recognize that constitutively activated defence responses can potentially result in a growth trade-off. Other future applications of CRISPR-mediated editing may retain active CRISPR systems, incorporating synthetic biology to balance trade-offs between modifying defence responses and safeguarding crop productivity. In addition to gene knockouts discussed previously, Section 6.1 below presents the use of CRISPR systems for more precise modifications and has potential for near-term impact. Sections 6.2–6.4 present broad future approaches for utilizing CRISPR systems to directly regulate host or pathogen response and defence, with implications for biological discovery. In all of these directions, the integrated implementation of emerging CRISPR technologies emerges as a promising avenue for advancing crop productivity.

### 6.1 Precise modification of amino acid sequence

Constitutive resistance responses can be engineered through gene knockout of negative regulators, though it may entail a growth trade-off. On the other hand, targeted defence necessitates precise modification of amino acid sequences on specific receptor-ligand binding sites or protein-protein interaction sites. Recognition of parasitic plant signals and effectors is the critical first step in host immunity. CRISPR base editors or prime editors offer a promising strategy to modify peptide sequences responsible for detecting pathogenic effectors while preserving signal transduction motifs (Ren et al., 2021; Zong et al., 2022). Flexible PAM base editors enhance the adaptability of this approach, allowing gRNA targeting to any codon of interest (Ren et al., 2021).

**FIGURE 2:**
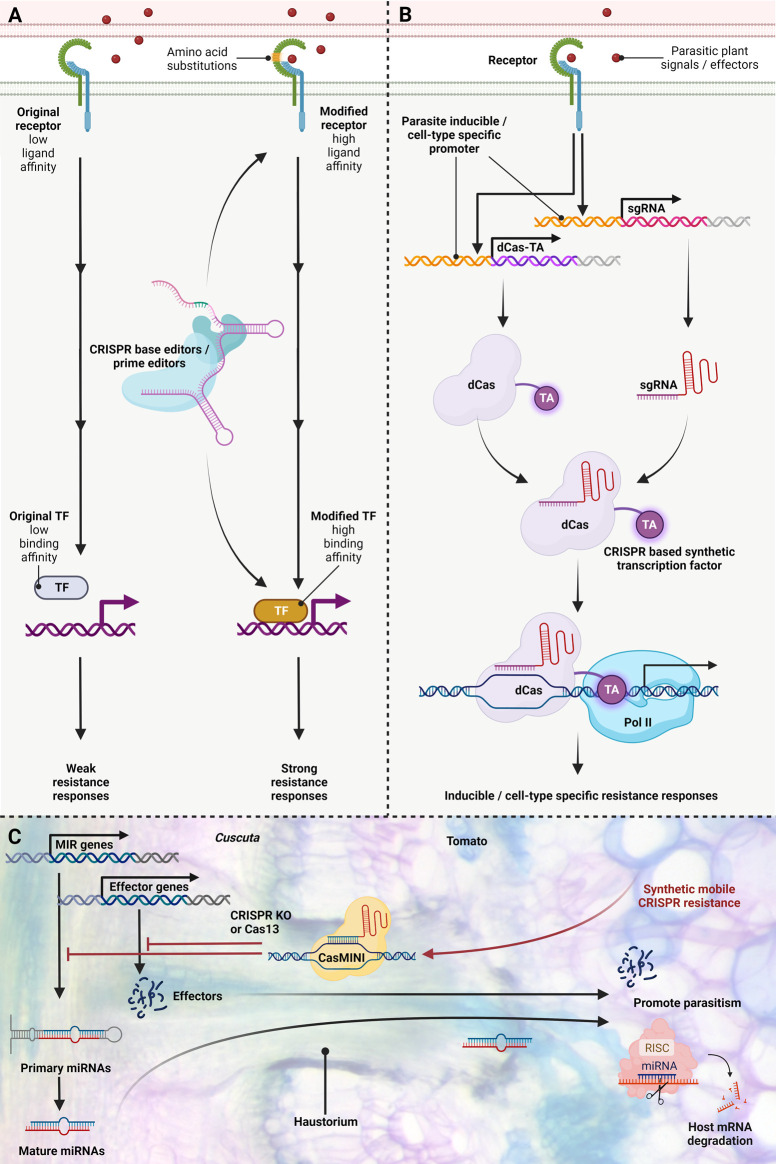
Enhancing Parasitic Plant Resistance using new CRISPR Technologies. **(A)** Protein engineering of receptors or transcription factors via CRISPR base and prime editing modifies parasite perception and protein binding affinity. Susceptibility of certain host plants to parasitic plants results from signal or effector non-recognition, hampering immune responses. CRISPR base and prime editing on receptors allows pathogen/effector perception, initiating defence signalling. In parallel, susceptibility in some host plants arises from the inability to activate downstream resistance due to a missing link in transcriptional activation. CRISPR base and prime editing adjusts transcription factor binding affinity, bridging connections and promoting downstream defence responses. **(B)** Conditional immunity with inducible or cell/tissue-specific activation via CRISPR-mediated transcriptional regulation. Inducible defence responses against parasitic plants are achieved through tailored promoters that express Cas enzymes and single-guide (sg) RNAs upon sensing parasitic signals or effectors. Inactive dCas enzymes are unable to cleave DNA but can still bind specific sequences via guide RNAs. dCas proteins fused with transcriptional activators (TA) trigger resistance-associated gene expression. Cell and tissue-type-specific promoters driving dCas enzymes and sgRNA expression can confer localized defence responses. Therefore, the activation of particular target genes can be directed with CRISPR-based synthetic transcription factor complexes. This CRISPR-mediated transcriptional regulation strategy offers conditionally activated transcription for parasitic plant resistance. **(C)** Hypothetical illustration of synthetic mobile CRISPR application for enhancing host resistance against parasitic plants. Based on previous studies, parasitic plants haustorium not only can transport water and nutrients but can also transport miRNA, mRNA, and small peptides bidirectionally, and these mobile *C. campestris* molecules might act as trans-species regulators of host-gene expression and may act as effectors or virulence factors to promote parasitism. CRISPR can be applied in plant host resistance by directly targeting genes of parasitic plants. Recent advancements offer compact CRISPR-Cas variants like CasΦ and CasMINI, under half the size of traditional Cas9. These compact forms could serve as candidates transported through haustoria to directly modulate parasitic plant genes. Leveraging CRISPR KO for targeted mutation and Cas13 for highly precise transcriptional regulation. This figure was created with https://www.biorender.com/.

The vulnerability of specific host plants to parasitic plants results from the failure to recognize signals or effectors, impeding effective immune responses (Hegenauer et al., 2020). For example, *CuRe1*, a typical LRR-RLP gene, is found in the genomic DNA of *S. lycopersicum* but is missing in *S. pennellii*, which is susceptible to *Cuscuta reflexa*. Notably, *CuRe1* in *S. lycopersicum* has two closely related genes, sharing 82% and 72% amino acid sequence similarity. However, these paralogs do not induce ethylene production when exposed to the *Cuscuta* factor. Similarly, other Solanaceae species, like *N. benthamiana* and *S. tuberosum*, have *CuRe1-like* genes with amino acid identities ranging from 70% to 80%, but also do not confer responsiveness to the *Cuscuta* factor in these species (Hegenauer et al., 2016). It's worth mentioning that within the 1121 amino acid length CuRe1 protein, there are only 18 potential N-glycosylation sites (NxT/S) in the LRR ectodomain (Hegenauer et al., 2016). These sites could play a pivotal role in protein folding and influence ligand binding. These NxT/S sites might serve as potential candidate targets for CRISPR base or prime editing to engineer receptor structure, potentially enhancing their ability to detect parasite signals or effectors and thus initiating resistance signalling (Figure 2A modified receptor). Similarly, susceptibility in certain host plants emerges from the incapacity to trigger downstream resistance due to a deficiency in transcriptional activation (Jhu et al., 2022a). In this context, CRISPR base or prime editing can fine-tune the binding affinity of transcription factors, bridging the gap and fostering subsequent defence reactions (Figure 2A modified TF).

### 6.2 Inducible defence responses

Inducible defence responses are an adaptive mechanism triggered by plants upon detecting threats such as pathogens, herbivores, or parasites. This mechanism optimizes resource allocation, thereby bolstering survival and reproductive success (Shudo and Iwasa, 2001). Prior research suggests many post-attachment resistance reactions against parasitic plants leverage inducible mechanisms that precisely activate in the presence of such parasites (Jhu et al., 2022a). This intricate host-parasitic plant interplay likely guides the co-evolution of resistance strategies, explaining the diverse gene expression profiles and resistance responses among different crop genotypes cultivated across various African regions (Kavuluko et al., 2021; Mutinda et al., 2023). Embracing inducible defence responses holds critical significance in genetic engineering and breeding endeavours geared towards developing improved future crops (Gurr and Rushton, 2005).

CRISPR technologies are well poised to enable inducible defence response. Expression of Cas enzymes by inducible promoters enables genome manipulation only in response to specific stimuli including pathogens and parasites (Ji et al., 2018; Wang et al., 2020). Of particular interest is the use of CRISPR/Cas-based artificial transcription factors in which Cas enzymes are tagged with enzymes repressing or promoting the transcription of a particular gene (Pan et al., 2021). Using multiplexed gRNA expression, entire pathways can be artificially regulated as an adaptive immunity mechanism. For example, inducible defence responses can be achieved by utilizing promoters that can be activated upon perceiving parasitic plant signals or effectors to drive the expression of Cas proteins and guide RNAs (Figure 2B). This CRISPR-based synthetic transcriptional regulation fuses a Cas protein to a transcriptional activator, which can then activate downstream genes involved in resistance responses (Figure 2B). This multifaceted approach to resistance enables broad-spectrum resistance, utilizes preexisting inducible multilayer resistance responses (Yoshida and Shirasu, 2009; Fishman and Shirasu, 2021; Jhu and Sinha, 2022) by expression of Cas from endogenous host promoters and will not be easily overcome by parasitic plants. Furthermore, inducible expression of CRISPR/Cas reagents reduces potential off-target or pleiotropic effects of defence response (Ji et al., 2018).

### 6.3 Cell-type or tissue-type specific defence mechanisms

Cell-type-specific barriers and defence mechanisms at the host and parasite interface constitute a pivotal aspect of plants’ repertoire to counteract parasitic plant incursions (Hu et al., 2020; Jhu et al., 2022b; Kawa and Brady, 2022). These mechanisms encompass diverse facets, such as epidermal barriers that physically redirect or impede parasitic plant structures, cortex barriers fortified with substances like lignin, or callose, and endodermal barriers fostering lignin, silica, or phenolic compound accumulations that thwart parasitic plant penetration (Yoshida and Shirasu, 2009; Yoder and Scholes, 2010; Mutuku et al., 2019). Such cell-type-specific defence mechanisms decisively curtail the invasion, establishment, and subsequent development of parasitic plants.

Similar to inducible defence response, cell-type-specific promoters can limit CRISPR activity to desired cell types (Decaestecker et al., 2019). Cell-types and tissue-types-specific promoters driving Cas enzymes and guide RNA expression can confer localized defence responses (Figure 2B). We anticipate the continued use of single-cell RNA sequencing technology (Cole et al., 2021; Cuperus, 2022) and spatial transcriptomics (Giacomello et al., 2017; Pour and Yanai, 2022) will facilitate the discovery of cell-type specific gene regulatory elements which can be exploited for genome engineering applications.

### 6.4 Direct targeting of parasitic plant genes and miRNAs

Based on prior research, haustoria of parasitic plants serve not only as conduits for water and nutrients but also facilitate the bidirectional transport of miRNA, mRNA, and small peptides (Kim et al., 2014; Shahid et al., 2018; Liu et al., 2020). Recent investigations have demonstrated inter-species small RNA trafficking through haustoria between *C. campestris* and its host and prompted the hypothesis that mobile miRNAs from *C. campestris* might function as cross-species regulators, influencing host gene expression and potentially acting as virulence factors that enhance parasitism (Figure 2C) (Shahid et al., 2018; Wu, 2018; Johnson and Axtell, 2019). On the other hand, multiple earlier studies have employed host-induced gene silencing (HIGS) to combat parasitic plants by generating transgenic host plants that produce specific small RNAs targeting genes of the parasitic plant (Tomilov et al., 2008; Alakonya et al., 2012; Farrokhi et al., 2019; Jhu et al., 2021; Jhu et al., 2022b). In a similar role, CRISPR may be applied for plant host resistance by directly targeting genes, mRNAs, and miRNAs of parasitic plants (Figure 2C). A distinct advantage of this approach is the ability to easily express multiplexed gRNAs to confer multilayer defence responses (Zhan et al., 2023). Indeed, multiplexed targeting of essential viral protein regions successfully reduced viral pathogen accumulation by 99% (Ji et al., 2018). Furthermore, by using inducible promoters this study was not able to detect statistically significant off-target mutations in transgenic plants (Ji et al., 2018). gRNAs designed for this approach should specifically recognize pivotal parasite effectors or virulence factors, including mobile miRNAs, while avoiding targets also present within the host plant. Harnessing Cas enzymes targeting RNA, such as Cas13 (Abudayyeh et al., 2017), allows highly specific and multiplexed regulation of parasitic plant gene expression at the transcriptional level. Utilizing multiple methods to directly regulate parasitic gene expression, including both CRISPR and proven HIGS targeting, is likely to be critical for broad spectrum, effective host immunity. A pivotal aspect of adopting this approach is the optimization of CRISPR reagents, ensuring enhanced mobility and high specificity. The foremost challenge revolves around delivering CRISPR/Cas components effectively. The widely utilized CRISPR Cas9, a 160-kDa protein (Jinek et al., 2014), poses delivery hurdles due to its substantial size. Notably, previous research indicates that the majority of mobile proteins transported via haustoria range from 20 to 70 kilodaltons (kDa), though a noteworthy 20% exceed 70 kDa, with the largest reaching 611 kDa (Liu et al., 2020). Moreover, technological advancements have yielded smaller alternatives such as CRISPR CasΦ or CasMINI (Pausch et al., 2020; Xu et al., 2021), each less than half the size of conventional Cas9. These compact Cas variants hold promise as potential candidates that can be transported via haustorium and target parasitic plant genes directly (Figure 2C). Also promising is the inclusion of mobile RNA motifs to promote cell to cell mobility of gene editing reagents (Ellison et al., 2020). Mobile RNAs have been utilized to move CRISPR reagents across graft junctions from transgenic rootstock to wild-type meristematic cells (Yang et al., 2023). Investigating transport mechanisms and incorporating mobile motifs into Cas proteins will be pivotal in future research directions to facilitate their transport.

## 7 Conclusion

In harnessing the potential of CRISPR technologies for enhanced crop protection, the intricate balance between modifying defence responses and preserving crop yield becomes apparent. Through high-throughput gene editing, targeted nucleotide modifications, and synthetic gene regulation, CRISPR systems have been shown to provide immense power in gene discovery and crop improvement. CRISPR knockout in bolstering pre-attachment resistance by targeting strigolactone pathways and enhancing post-attachment defences through cell wall fortification offers promising avenues for combating parasitic plants. However, the trade-offs of genetic modifications impacting plant growth and physiology, underline the need for precise regulatory approaches. Inducible defence responses through innovative synthetic transcriptional regulation offer adaptive immunity, while cell-type specificity empowers localized defences. The precise modification of amino acid sequences using CRISPR base and prime editing presents a future of tailored immunity. The convergence of these strategies embodies a promising avenue for bolstering crop productivity and resilience, underpinning a transformative shift in agricultural practices towards more robust and sustainable solutions.

## References

[B1] Abdullahi W. M. Dianda M. Boukar O. Dieng I. Mohammed G. S. Belko N. (2022). Integrated management of Striga gesnerioides in cowpea using resistant varieties, improved crop nutrition and rhizobium inoculants. Plant Soil 473, 197–213. 10.1007/s11104-022-05295-7

[B2] Abudayyeh O. O. Gootenberg J. S. Essletzbichler P. Han S. Joung J. Belanto J. J. (2017). RNA targeting with CRISPR-Cas13. Nature 550, 280–284. 10.1038/nature24049 28976959 PMC5706658

[B3] Adewale S. A. Badu-Apraku B. Akinwale R. O. Paterne A. A. Gedil M. Garcia-Oliveira A. L. (2020). Genome-wide association study of Striga resistance in early maturing white tropical maize inbred lines. BMC Plant Biol. 20, 203. 10.1186/s12870-020-02360-0 32393176 PMC7212567

[B4] Alakonya A. Kumar R. Koenig D. Kimura S. Townsley B. Runo S. (2012). Interspecific RNA interference of SHOOT MERISTEMLESS-like disrupts Cuscuta pentagona plant parasitism. Plant Cell 24, 3153–3166. 10.1105/tpc.112.099994 22822208 PMC3426138

[B5] Alder A. Jamil M. Marzorati M. Bruno M. Vermathen M. Bigler P. (2012). The path from β-carotene to carlactone, a strigolactone-like plant hormone. Science 335, 1348–1351. 10.1126/science.1218094 22422982

[B6] Bari V. K. Nassar J. A. Aly R. (2021). CRISPR/Cas9 mediated mutagenesis of More Axillary Growth 1 in tomato confers resistance to root parasitic weed Phelipanche aegyptiaca. Sci. Rep. 11, 3905. 10.1038/s41598-021-82897-8 33594101 PMC7887253

[B7] Bellis E. S. Kelly E. A. Lorts C. M. Gao H. DeLeo V. L. Rouhan G. (2020). Genomics of sorghum local adaptation to a parasitic plant. Proc. Natl. Acad. Sci. 117, 4243–4251. 10.1073/pnas.1908707117 32047036 PMC7049153

[B8] Boubakri H. (2023). Recent progress in CRISPR/Cas9-based genome editing for enhancing plant disease resistance. Gene 866, 147334. 10.1016/j.gene.2023.147334 36871676

[B9] Capdeville N. Schindele P. Puchta H. (2023). Getting better all the time — recent progress in the development of CRISPR/Cas-based tools for plant genome engineering. Curr. Opin. Biotechnol. 79, 102854. 10.1016/J.COPBIO.2022.102854 36455451

[B10] Chaloner T. M. Gurr S. J. Bebber D. P. (2021). Plant pathogen infection risk tracks global crop yields under climate change. Nat. Clim. Chang. 11, 710–715. 10.1038/s41558-021-01104-8

[B11] Che P. Wu E. Simon M. K. Anand A. Lowe K. Gao H. (2022). Wuschel2 enables highly efficient CRISPR/Cas-targeted genome editing during rapid *de novo* shoot regeneration in sorghum. Commun. Biol. 5, 344. 10.1038/s42003-022-03308-w 35410430 PMC9001672

[B12] Cole B. Bergmann D. Blaby-Haas C. E. Blaby I. K. Bouchard K. E. Brady S. M. (2021). Plant single-cell solutions for energy and the environment. Commun. Biol. 4, 962. 10.1038/s42003-021-02477-4 34385583 PMC8361165

[B13] Cuperus J. T. (2022). Single-cell genomics in plants: current state, future directions, and hurdles to overcome. Plant Physiol. 188, 749–755. 10.1093/plphys/kiab478 34662424 PMC8825463

[B14] Dangl J. L. Horvath D. M. Staskawicz B. J. (2013). Pivoting the plant immune system from dissection to deployment. Science 341, 746–751. 10.1126/science.1236011 23950531 PMC3869199

[B15] David O. G. Ayangbenro A. S. Odhiambo J. J. O. Babalola O. O. (2022). Striga hermonthica: a highly destructive pathogen in maize production. Environ. Challenges 8, 100590. 10.1016/j.envc.2022.100590

[B16] Decaestecker W. Buono R. A. Pfeiffer M. L. Vangheluwe N. Jourquin J. Karimi M. (2019). CRISPR-TSKO: a technique for efficient mutagenesis in specific cell types, tissues, or organs in arabidopsis. Plant Cell 31, 2868–2887. 10.1105/tpc.19.00454 31562216 PMC6925012

[B17] Demirer G. S. Zhang H. Goh N. S. Pinals R. L. Chang R. Landry M. P. (2023). Carbon nanocarriers deliver siRNA to intact plant cells for efficient gene knockdown. Sci. Adv. 6, eaaz0495. 10.1126/sciadv.aaz0495 PMC7314522 32637592

[B18] Echevarría-Zomeño S. Pérez-De-Luque A. Jorrín J. Maldonado A. M. (2006). Pre-haustorial resistance to broomrape (Orobanche cumana) in sunflower (Helianthus annuus): cytochemical studies. J. Exp. Bot. 57, 4189–4200. 10.1093/jxb/erl195 17095573

[B19] Ejeta G. (2007). “The striga scourge in Africa: a growing pandemic,” in Integrating new technologies for Striga control (Singapore: World Scientific), 3–16. 10.1142/9789812771506_0001

[B20] Ellison E. E. Nagalakshmi U. Gamo M. E. Huang P. Dinesh-Kumar S. Voytas D. F. (2020). Multiplexed heritable gene editing using RNA viruses and mobile single guide RNAs. Nat. Plants 6, 620–624. 10.1038/s41477-020-0670-y 32483329

[B21] Farrokhi Z. Alizadeh H. Alizadeh H. Mehrizi F. A. (2019). Host-induced silencing of some important genes involved in osmoregulation of parasitic plant Phelipanche aegyptiaca. Mol. Biotechnol. 61, 929–937. 10.1007/s12033-019-00215-0 31564035

[B22] Fishman M. R. Shirasu K. (2021). How to resist parasitic plants: pre- and post-attachment strategies. Curr. Opin. Plant Biol. 62, 102004. 10.1016/j.pbi.2021.102004 33647828

[B23] Gaillochet C. Develtere W. Jacobs T. B. (2021). CRISPR screens in plants: approaches, guidelines, and future prospects. Plant Cell 33, 794–813. 10.1093/plcell/koab099 33823021 PMC8226290

[B24] Gao C. (2021). Genome engineering for crop improvement and future agriculture. Cell 184, 1621–1635. 10.1016/J.CELL.2021.01.005 33581057

[B25] Gerbore J. Benhamou N. Vallance J. Le Floch G. Grizard D. Regnault-Roger C. (2014). Biological control of plant pathogens: advantages and limitations seen through the case study of Pythium oligandrum. Environ. Sci. Pollut. Res. 21, 4847–4860. 10.1007/s11356-013-1807-6 23695856

[B26] Giacomello S. Salmén F. Terebieniec B. K. Vickovic S. Navarro J. F. Alexeyenko A. (2017). Spatially resolved transcriptome profiling in model plant species. Nat. Plants 3, 17061. 10.1038/nplants.2017.61 28481330

[B27] Gobena D. Shimels M. Rich P. J. Ruyter-Spira C. Bouwmeester H. Kanuganti S. (2017). Mutation in sorghum LOW GERMINATION STIMULANT 1 alters strigolactones and causes Striga resistance. Proc. Natl. Acad. Sci. 114, 4471–4476. 10.1073/pnas.1618965114 28396420 PMC5410831

[B28] Gurr S. J. Rushton P. J. (2005). Engineering plants with increased disease resistance: what are we going to express? Trends Biotechnol. 23, 275–282. 10.1016/j.tibtech.2005.04.007 15922079

[B29] Hegenauer V. Fürst U. Kaiser B. Smoker M. Zipfel C. Felix G. (2016). Detection of the plant parasite Cuscuta reflexa by a tomato cell surface receptor. Science 353, 478–481. 10.1126/science.aaf3919 27471302

[B30] Hegenauer V. Slaby P. Körner M. Bruckmüller J. A. Burggraf R. Albert I. (2020). The tomato receptor CuRe1 senses a cell wall protein to identify Cuscuta as a pathogen. Nat. Commun. 11, 5299. 10.1038/s41467-020-19147-4 33082345 PMC7576778

[B31] Hu L. Wang J. Yang C. Islam F. Bouwmeester H. J. Muños S. (2020). The effect of virulence and resistance mechanisms on the interactions between parasitic plants and their hosts. Int. J. Mol. Sci. 21, 9013–9027. 10.3390/ijms21239013 33260931 PMC7730841

[B32] Jhu M.-Y. Sinha N. R. (2022). Parasitic plants: an Overview of mechanisms by which plants perceive and respond to parasites. Annu. Rev. Plant Biol. 73, 433–455. 10.1146/annurev-arplant-102820-100635 35363532

[B33] Jhu M. Y. Farhi M. Wang L. Philbrook R. N. Belcher M. S. Nakayama H. (2022a). Heinz-resistant tomato cultivars exhibit a lignin-based resistance to field dodder (Cuscuta campestris) parasitism. Plant Physiol. 189, 129–151. 10.1093/plphys/kiac024 35099559 PMC9070836

[B34] Jhu M. Y. Farhi M. Wang L. Zumstein K. Sinha N. R. (2022b). Investigating host and parasitic plant interaction by tissue-specific gene analyses on tomato and Cuscuta campestris interface at three haustorial developmental stages. Front. Plant Sci. 12, 764843. 10.3389/fpls.2021.764843 35222447 PMC8866705

[B35] Jhu M. Y. Ichihashi Y. Farhi M. Wong C. Sinha N. R. (2021). Lateral Organ Boundaries Domain 25 functions as a key regulator of haustorium development in dodders. Plant Physiol. 186, 2093–2110. 10.1093/plphys/kiab231 34618110 PMC8331169

[B36] Ji X. Si X. Zhang Y. Zhang H. Zhang F. Gao C. (2018). Conferring DNA virus resistance with high specificity in plants using virus-inducible genome-editing system. Genome Biol. 19, 197. 10.1186/s13059-018-1580-4 30442181 PMC6238286

[B37] Jinek M. Chylinski K. Fonfara I. Hauer M. Doudna J. A. Charpentier E. (2012). A programmable dual-RNA–guided DNA endonuclease in adaptive bacterial immunity. Science 337, 816–821. 10.1126/science.1225829 22745249 PMC6286148

[B38] Jinek M. Jiang F. Taylor D. W. Sternberg S. H. Kaya E. Ma E. (2014). Structures of Cas9 endonucleases reveal RNA-mediated conformational activation. Science 343, 1247997. 10.1126/science.1247997 24505130 PMC4184034

[B39] Johnson N. R. Axtell M. J. (2019). Small RNA warfare: exploring origins and function of trans-species microRNAs from the parasitic plant Cuscuta. Curr. Opin. Plant Biol. 50, 76–81. 10.1016/j.pbi.2019.03.014 31029811

[B40] Kanampiu F. Makumbi D. Mageto E. Omanya G. Waruingi S. Musyoka P. (2018). Assessment of management options on striga infestation and maize grain yield in Kenya. Weed Sci. 66, 516–524. 10.1017/wsc.2018.4 33583963 PMC7797635

[B41] Karmakar S. Das P. Panda D. Xie K. Baig M. J. Molla K. A. (2022). A detailed landscape of CRISPR-Cas-mediated plant disease and pest management. Plant Sci. 323, 111376. 10.1016/J.PLANTSCI.2022.111376 35835393

[B42] Kavuluko J. Kibe M. Sugut I. Kibet W. Masanga J. Mutinda S. (2021). GWAS provides biological insights into mechanisms of the parasitic plant (Striga) resistance in sorghum. BMC Plant Biol. 21, 392. 10.1186/s12870-021-03155-7 34418971 PMC8379865

[B43] Kawa D. Brady S. M. (2022). Root cell types as an interface for biotic interactions. Trends Plant Sci. 27, 1173–1186. 10.1016/j.tplants.2022.06.003 35792025

[B44] Kim G. LeBlanc M. L. Wafula E. K. dePamphilis C. W. Westwood J. H. (2014). Plant science. Genomic-scale exchange of mRNA between a parasitic plant and its hosts. Science 345, 808–811. 10.1126/science.1253122 25124438

[B45] Kodama K. Rich M. K. Yoda A. Shimazaki S. Xie X. Akiyama K. (2022). An ancestral function of strigolactones as symbiotic rhizosphere signals. Nat. Commun. 13, 3974. 10.1038/s41467-022-31708-3 35803942 PMC9270392

[B46] Langner T. Kamoun S. Belhaj K. (2018). CRISPR crops: plant genome editing toward disease resistance. Annu. Rev. Phytopathol. 56, 479–512. 10.1146/annurev-phyto-080417-050158 29975607

[B47] Lanini W. T. Kogan M. (2005). Biology and management of Cuscuta in crops. Int. J. Agric. Nat. Resour. 32, 127–141. 10.7764/rcia.v32i3.317

[B48] Li J. Timko M. P. (2009). Gene-for-Gene resistance in striga-cowpea associations. Science 325, 1094. 10.1126/science.1174754 19713520

[B49] Liu N. Shen G. Xu Y. Liu H. Zhang J. Li S. (2020). Extensive inter-plant protein transfer between Cuscuta parasites and their host plants. Mol. Plant 13, 573–585. 10.1016/j.molp.2019.12.002 31812691

[B50] Maher M. F. Nasti R. A. Vollbrecht M. Starker C. G. Clark M. D. Voytas D. F. (2020). Plant gene editing through *de novo* induction of meristems. Nat. Biotechnol. 38, 84–89. 10.1038/s41587-019-0337-2 31844292 PMC6954279

[B51] Mayzlish-Gati E. LekKala S. P. Resnick N. Wininger S. Bhattacharya C. Lemcoff J. H. (2010). Strigolactones are positive regulators of light-harvesting genes in tomato. J. Exp. Bot. 61, 3129–3136. 10.1093/jxb/erq138 20501744 PMC2892153

[B52] Menz J. Modrzejewski D. Hartung F. Wilhelm R. Sprink T. (2020). Genome edited crops touch the market: a view on the global development and regulatory environment. Front. Plant Sci. 11, 586027. 10.3389/fpls.2020.586027 33163013 PMC7581933

[B53] Mutinda S. Mobegi F. M. Hale B. Dayou O. Ateka E. Wijeratne A. (2023). Resolving intergenotypic Striga resistance in sorghum. J. Exp. Bot. 74, 5294–5306. 10.1093/jxb/erad210 37260405 PMC10498017

[B54] Mutuku J. M. Cui S. Hori C. Takeda Y. Tobimatsu Y. Nakabayashi R. (2019). The structural integrity of lignin is crucial for resistance against Striga hermonthica parasitism in rice. Plant Physiol. 179, 1796–1809. 10.1104/pp.18.01133 30670602 PMC6446757

[B55] Pour M. Yanai I. (2022). New adventures in spatial transcriptomics. Dev. Cell 57, 1209–1210. 10.1016/j.devcel.2022.04.021 35609527

[B56] Pan C. Sretenovic S. Qi Y. (2021). CRISPR/dCas-mediated transcriptional and epigenetic regulation in plants. Curr. Opin. Plant Biol. 60, 101980. 10.1016/J.PBI.2020.101980 33401227

[B57] Pausch P. Al-Shayeb B. Bisom-Rapp E. Tsuchida C. A. Li Z. Cress B. F. (2020). CRISPR-CasΦ from huge phages is a hypercompact genome editor. Science 369, 333–337. 10.1126/science.abb1400 32675376 PMC8207990

[B58] Pixley K. V. Falck-Zepeda J. B. Paarlberg R. L. Phillips P. W. B. Slamet-Loedin I. H. Dhugga K. S. (2022). Genome-edited crops for improved food security of smallholder farmers. Nat. Genet. 54, 364–367. 10.1038/s41588-022-01046-7 35393597

[B59] Ren Q. Sretenovic S. Liu S. Tang X. Huang L. He Y. (2021). PAM-less plant genome editing using a CRISPR–SpRY toolbox. Nat. Plants 7, 25–33. 10.1038/s41477-020-00827-4 33398158

[B60] Rich P. J. Grenier C. Ejeta G. (2004). Striga resistance in the wild relatives of sorghum. Crop Sci. 44, 2221–2229. 10.2135/cropsci2004.2221

[B61] Rodenburg J. Demont M. Zwart S. J. Bastiaans L. (2016). Parasitic weed incidence and related economic losses in rice in Africa. Agric. Ecosyst. Environ. 235, 306–317. 10.1016/j.agee.2016.10.020

[B62] Rodríguez-Leal D. Lemmon Z. H. Man J. Bartlett M. E. Lippman Z. B. (2017). Engineering quantitative trait variation for crop improvement by genome editing. Cell 171, 470–480. 10.1016/J.CELL.2017.08.030 28919077

[B63] Sauerborn J. Müller-Stöver D. Hershenhorn J. (2007). The role of biological control in managing parasitic weeds. Crop Prot. 26, 246–254. 10.1016/j.cropro.2005.12.012

[B64] Savary S. Willocquet L. Pethybridge S. J. Esker P. McRoberts N. Nelson A. (2019). The global burden of pathogens and pests on major food crops. Nat. Ecol. Evol. 3, 430–439. 10.1038/s41559-018-0793-y 30718852

[B65] Serghini K. de Luque A. P. Castejón-Muñoz M. García-Torres L. Jorrín J. V. (2001). Sunflower (Helianthus annuus L.) response to broomrape (Orobanche cernua Loefl.) parasitism: induced synthesis and excretion of 7-hydroxylated simple coumarins. J. Exp. Bot. 52, 2227–2234. 10.1093/jexbot/52.364.2227 11604462

[B66] Seto Y. Sado A. Asami K. Hanada A. Umehara M. Akiyama K. (2014). Carlactone is an endogenous biosynthetic precursor for strigolactones. Proc. Natl. Acad. Sci. U. S. A. 111, 1640–1645. 10.1073/pnas.1314805111 24434551 PMC3910621

[B67] Shahid S. Kim G. Johnson N. R. Wafula E. Wang F. Coruh C. (2018). MicroRNAs from the parasitic plant Cuscuta campestris target host messenger RNAs. Nature 553, 82–85. 10.1038/nature25027 29300014

[B68] Shudo E. Iwasa Y. (2001). Inducible defense against pathogens and parasites: optimal choice among multiple options. J. Theor. Biol. 209, 233–247. 10.1006/jtbi.2000.2259 11401465

[B69] Su C. Liu H. Wafula E. K. Honaas L. de Pamphilis C. W. Timko M. P. (2020). SHR4z, a novel decoy effector from the haustorium of the parasitic weed Striga gesnerioides, suppresses host plant immunity. New Phytol. 226, 891–908. 10.1111/nph.16351 31788811 PMC7187149

[B70] Tester M. Langridge P. (2010). Breeding technologies to increase crop production in a changing world. Science 327, 818–822. 10.1126/science.1183700 20150489

[B71] Toker C. Yadav S. S. Solanki I. S. (2007). “Mutation breeding,” in Lentil: an ancient crop for modern times. Editors Yadav S. S. McNeil D. L. Stevenson P. C. (Dordrecht: Springer Netherlands), 209–224. 10.1007/978-1-4020-6313-8_13

[B72] Tomilov A. A. Tomilova N. B. Wroblewski T. Michelmore R. Yoder J. I. (2008). Trans-specific gene silencing between host and parasitic plants. Plant J. 56, 389–397. 10.1111/j.1365-313X.2008.03613.x 18643992

[B73] van Schie C. C. N. Takken F. L. W. (2014). Susceptibility genes 101: how to Be a good host. Annu. Rev. Phytopathol. 52, 551–581. 10.1146/annurev-phyto-102313-045854 25001453

[B74] Veillet F. Durand M. Kroj T. Cesari S. Gallois J. L. (2020). Precision breeding made real with CRISPR: illustration through genetic resistance to pathogens. Plant Commun. 1, 100102. 10.1016/j.xplc.2020.100102 33367260 PMC7747970

[B75] Wang X. Ye L. Lyu M. Ursache R. Löytynoja A. Mähönen A. P. (2020). An inducible genome editing system for plants. Nat. Plants 6, 766–772. 10.1038/s41477-020-0695-2 32601420 PMC7611339

[B76] Waters M. T. Gutjahr C. Bennett T. Nelson D. C. (2017). Strigolactone signaling and evolution. Annu. Rev. Plant Biol. 68, 291–322. 10.1146/annurev-arplant-042916-040925 28125281

[B77] Wu J. (2018). miRNAs as a secret weapon in the battlefield of haustoria, the interface between parasites and host plants. Mol. Plant 11, 354–356. 10.1016/j.molp.2018.02.004 29462721

[B78] Xu X. Chemparathy A. Zeng L. Kempton H. R. Shang S. Nakamura M. (2021). Engineered miniature CRISPR-Cas system for mammalian genome regulation and editing. Mol. Cell 81, 4333–4345.e4. 10.1016/j.molcel.2021.08.008 34480847

[B79] Yang L. Machin F. Wang S. Saplaoura E. Kragler F. (2023). Heritable transgene-free genome editing in plants by grafting of wild-type shoots to transgenic donor rootstocks. Nat. Biotechnol. 41, 958–967. 10.1038/s41587-022-01585-8 36593415 PMC10344777

[B80] Yin K. Qiu J. L. (2019). Genome editing for plant disease resistance: applications and perspectives. Philosophical Trans. R. Soc. B Biol. Sci. 374, 20180322. 10.1098/rstb.2018.0322 PMC6367152 30967029

[B81] Yoder J. I. Scholes J. D. (2010). Host plant resistance to parasitic weeds; recent progress and bottlenecks. Curr. Opin. Plant Biol. 13, 478–484. 10.1016/j.pbi.2010.04.011 20627804

[B82] Yoneyama K. Awad A. A. Xie X. Yoneyama K. Takeuchi Y. (2010). Strigolactones as germination stimulants for root parasitic plants. Plant Cell Physiol. 51, 1095–1103. 10.1093/pcp/pcq055 20403809 PMC2900819

[B83] Yoshida S. Cui S. Ichihashi Y. Shirasu K. (2016). The haustorium, a specialized invasive organ in parasitic plants. Annu. Rev. Plant Biol. 67, 643–667. 10.1146/annurev-arplant-043015-111702 27128469

[B84] Yoshida S. Shirasu K. (2009). Multiple layers of incompatibility to the parasitic witchweed, Striga hermonthica. New Phytol. 183, 180–189. 10.1111/j.1469-8137.2009.02840.x 19402875

[B85] Zaidi S. S. Mahas A. Vanderschuren H. Mahfouz M. M. (2020). Engineering crops of the future: CRISPR approaches to develop climate-resilient and disease-resistant plants. Genome Biol. 21, 289. 10.1186/s13059-020-02204-y 33256828 PMC7702697

[B86] Zhan X. Liu W. Nie B. Zhang F. Zhang J. (2023). Cas13d-mediated multiplex RNA targeting confers a broad-spectrum resistance against RNA viruses in potato. Commun. Biol. 6, 855. 10.1038/s42003-023-05205-2 37591976 PMC10435558

[B87] Zhang Y. Ren Q. Tang X. Liu S. Malzahn A. A. Zhou J. (2021). Expanding the scope of plant genome engineering with Cas12a orthologs and highly multiplexable editing systems. Nat. Commun. 12, 1944. 10.1038/s41467-021-22330-w 33782402 PMC8007695

[B88] Zong Y. Liu Y. Xue C. Li B. Li X. Wang Y. (2022). An engineered prime editor with enhanced editing efficiency in plants. Nat. Biotechnol. 40, 1394–1402. 10.1038/s41587-022-01254-w 35332341

[B89] Zsögön A. Čermák T. Naves E. R. Notini M. M. Edel K. H. Weinl S. (2018). *De novo* domestication of wild tomato using genome editing. Nat. Biotechnol. 36, 1211–1216. 10.1038/nbt.4272 30272678

